# Listen to Us: Perceptions of Animal Voice and Agency

**DOI:** 10.3390/ani13203271

**Published:** 2023-10-19

**Authors:** Anja M. Thomsen, William T. Borrie, Kelly K. Miller, Adam P. A. Cardilini

**Affiliations:** 1 School of Life and Environmental Sciences, Deakin University, Melbourne, VIC 3125, Australia; b.borrie@deakin.edu.au (W.T.B.); kelly.miller@deakin.edu.au (K.K.M.); adam.cardilini@deakin.edu.au (A.P.A.C.); 2 PAN Works, Marlborough, MA 01752, USA

**Keywords:** animal representation, animal communication, human perceptions, human language constructs and paradigms, human–animal relationships, *animal voice* amplification

## Abstract

**Simple Summary:**

In this study, we used open-ended interviews to explore how animal moral claims and interests may be best represented in the human world. The findings suggest that the key to animal representation lies in human perceptions of *animal voice* and that these perceptions are shaped by cultural, social, economic, legal, and political language constructs and paradigms. Our findings illustrate how the human contextual definition of animals as voiceless or as having a voice has serious implications for animals, society, and the environment. This study highlights the importance of recognising *animal voice* as crucial for animal representation and draws parallels with similar calls in the literature. We recommend future research to focus on developing ethical, compassionate, and respectful approaches to understanding animal subjective experiences to empower and amplify animal voices.

**Abstract:**

In many contexts, the interests of nonhuman animals (hereafter “animals”) are often overlooked or considered to be a lower priority than those of humans. While strong arguments exist for taking animal moral claims seriously, these largely go unheard due to dominant anthropocentric attitudes and beliefs. This study aimed to explore how animal interests might be best represented in the human world. We conducted interviews to investigate people’s perceptions of what it means to speak for other animals and who can reliably represent animal interests. Using Grounded Theory analytical methods, we identified one major theme: “Animal voice”, and its subthemes: “Animals do/do not have a voice”, “Human language constructs realities and paradigms”, and “Let animals speak”. Our findings illustrate how human language constructs contribute to shaping the realities of animals by contextually defining them as voiceless. This has serious implications for animals, society, and the environment. Drawing parallels with the relevant literature, our results reflect calls for the social and political recognition of *animal voice* as fundamental to animal representation. We recommend future research to focus on developing ethical and compassionate approaches to understanding animal subjective experiences to empower and amplify animal voices.

## 1. Introduction

In many contexts, the needs and wishes of nonhuman animals (hereafter animals) are often overlooked or considered to be a lower priority than those of humans. This has significant ethical, social, cultural, environmental, economic, and political implications, with growing public and academic concerns over how animals are considered in society [1,2,3,4,5,6,7,8]. Historical and predominantly Western philosophical ideas and constructs have contributed to a general acceptance of human animal superiority over other animals [9]. Perhaps the most extreme expression of these ideas, animals as “automata” (machines), was conceptualised by influential French philosopher Renee Descartes 1637, arguably absolving humans of moral responsibility towards other animals [9,10]. Both Descartes and the Enlightenment philosopher Immanuel Kant defended the human/animal distinction based on the assumption that non-human animals do not possess a mind and, therefore, lack self-awareness and the ability to make themselves understood through language. However, prominent thinkers have also criticised the human/animal distinction and that this distinction was enough to exclude animals from moral consideration. Jeremy Bentham famously summarised their critical stance: “The question is not, Can they reason? Nor, Can they talk? But, Can they suffer?” [11]. In a similar critique, Henry S. Salt (1894) was one of the first to challenge the human/animal dichotomy with an explicit focus on connecting animal rights to social progress [12].

The idea that animals lack rationality and mind, and that this distinction justifies the depersonalisation and commodification of animals as resources for humans, prevails in certain scientific, economic, social, and political systems today [13]. Conditions for most animals, both wild and domestic, have worsened in the last two centuries due to the intensification of both terrestrial and marine factory farming [14,15,16,17,18,19,20], the increasing destruction and disturbance of wild animal habitats for urban and agricultural land development [21,22,23,24,25], and the impacts of climate change such as wildfires, desertification, ocean acidification, rising nutrient loads in water bodies, and more frequent extreme weather events [26,27,28,29,30].

Developing scientific research of animal cognitive, emotional, social, and cultural ability and complexity is increasingly challenging how we think about animal minds and lives [13,31]. For example, Broom (2003) described the widespread biased assumption that the only significant evolutionary path was that which leads to man, particularly as concerns brain function, as biologically naïve [32] (p. 85). Larger brains may store more memory, but this does not necessarily imply more cognitive ability [33].

Research has documented that, like us, animals seek comfort, playtime, companionship, freedom of expression, and display a variety of emotions and differences in personality [34,35,36,37,38,39,40,41,42,43,44,45,46,47]. Questioning the notion that culture is unique to humans, research also shows that animals have their own unique cultures and social structures where knowledge and language are passed on via generational learning [48,49,50,51,52,53,54]. Furthermore, an increasing body of studies have showed that emotional states, as observed in vertebrates, some cephalopods [55,56], and invertebrates [57], affect judgement and determine decision-making processes, as with humans [58,59,60,61]. Researchers have also called for the recognition of sentience in many animal groups we have not previously considered, most notably invertebrates [62]. For example, new research demonstrates that wasps can recognise species-specific individual faces, demonstrating evolutionary convergence between mammals and wasps, despite their differing sensory and neural structures [63], while other evidence has suggested hedonistic behaviour in bees [64]. Additionally, there are calls to improve our understanding of animal communication, cognition, subjective experience, agency, interests, and their place in society [65,66,67,68,69]. This research challenges old assumptions about animals and makes us question the human/animal dichotomy.

We must rethink our relations and entangled existences with animals if animals are to have dignified and flourishing lives. While animal advocacy has contributed to increasing public awareness about the issues confronting animals, its influence and impact has been limited [70]. While strong arguments exist for taking animal moral claims seriously, these largely go unheard because of inequalities in representation, similar to marginalised groups of humans in society [71]. Some authors therefore now argue for expanding the discussion of animal consideration beyond the typical discourses regarding *animal legal rights* or *duties towards animals*, to include the political representation of animals’ interests [72]. In the political and philosophical disciplines, this has been identified as part of the *Political Turn in Animal Ethics* [72,73].

Scholars have increasingly called for considering animal representation in social and political discourse [71,73,74,75,76,77,78,79,80,81,82,83,84,85,86], but many challenges remain in terms of how this could be realised. Magaña (2022) summarised these challenges by what they describe as *individual factors*—the aspects of human psychological mechanisms that predispose our thinking and behaviour in favour of the human perspective and interests. And *institutional factors* which relate to animals’ inability to vote, human political and economic interests to favour animal exploitation, and electoral barriers whereby, for example, minor pro-animal parties have limited political presence and influence [72]. However, animal representation currently faces the significant barrier of requiring humans to act as representatives on their behalf, which begs the question: How can we reliably represent the interests of animals, and who can do it? As such, one critical step for animal representation is to identify the characteristics of someone who can reliably speak for animal interests. This furthermore requires that we investigate what it means to reliably represent animals. The following question broadly addresses the issue: What does it mean to speak for animals?

To speak for anyone else, even other humans, is a difficult task. For example, certain groups in society may not be well represented in the political process including, but not limited to, people with disabilities, prisoners, the elderly, social housing clients, asylum seekers, the homeless, the unemployed, and other similarly vulnerable members of society [87]. Similar issues exist for animals, who may be considered particularly vulnerable in the sense that they are subject to laws imposed on them by humans, often reflecting the interests of the humans rather than those of the animals themselves [88]. In the human world, we have attempted to protect vulnerable members of society via various legal means. For example, like animals, human children are considered vulnerable in the sense that they may not be able to speak for themselves or protect themselves in specific contexts. In International Law, specific notions have therefore been developed to include particularly vulnerable individuals who may be considered unable to speak for themselves. The 1989 International Convention on the Rights of the Child therefore incorporated the dimension of vulnerability as a more precise criterion for inclusion because it extended to also include children with disabilities [89]. It may be productive to think about treating the issue of animal representation similarly.

Additionally, speaking for animals is already a challenge because animals communicate differently to humans. As such, because our systems are built on human language, communication, and interests, animals who communicate differently and have different interests fall outside this system [90,91]. And, while science can give us some of the practical tools to better understand how animals communicate their interests, there are still significant issues of interpretation.

One major challenge has been the historical focus on verbal language as the only meaningful form of communication. The logic of typical language models is flawed because it has been using human signalling behaviour as the benchmark for understanding all other forms of communication [92]. Expanding our understanding of what counts as language challenges the anthropocentric notion that difference is equal to deficiency and that we ought to consider differences as a matter of degree, not of kind [91]. However, it also challenges us to carefully consider differences to avoid anthropocentric misappropriations and misrepresentations in our attempts to understand and engage with animals [93]. For the sake of clarity, we emphasise that our use of the terms “animal communication”, “animal voice”, and “interspecies communication” implicitly acknowledge these challenges. We have therefore structured our discussion and the concept of *animal voice* from the viewpoint of Donna Haraway’s (2003) ethical approach, captured as humans and nonhumans “bonded in significant otherness” [94] (p. 16). The benefit of the ethical approach is that it can be extended to include cognitive, psychological, ethological, interactional (concerning “the nature of communication and the structure of interaction” [93] (p. 359)), and ontological approaches to understanding animal communication. As Kulick (2017) summarises, the ethical approach implies respectful and compassionate engagement with animals that avoids reducing their communication to anthropocentric projections or perpetuating cartesian notions of animals as exploitable “automata”. It furthermore acknowledges that recognising and understanding animal communication (for example, “through acts of protest and dissent” [90] (p. 31)) may as much be a matter of respectful non-engagement (e.g., [90,95]).

The aim of this study was to explore the issue of how animal interests might be best represented in the human world. Our approach was to capture thoughts from both experts in relevant academic fields, but also people with expertise and experience working with (e.g., farmers) and for (e.g., politicians, animal advocates) animals outside academia who can describe animal representation across multiple dimensions and identify gaps and challenges to better representing animals in the social–political arena. We conducted qualitative interviews with people who had significant experience with animals and/or issues relating to animals. This included people from a wide range of animal-related work and activities with various relationships to animals. We documented participants perceptions of animal representation by broadly asking: “Who can speak for animals?” Using a data-driven approach, we conducted thematic analysis to help identify and expand theoretical concepts related to animal representation. We compared and contrasted our results with the discussion being had in the literature to identify convergence, gaps, and future opportunities.

## 2. Materials and Methods

For this study, we conducted interviews to gather information related to people’s perceptions of what it means to speak for other animals and who can reliably represent animal interests. The semi-structured interviews were guided via methodologies in Grounded Theory and Thematic Analysis [96,97,98]. This qualitative approach is suited to poorly understood, complex, and/or emerging issues, or issues that require an in-depth scrutiny of untested assumptions [99,100]. We used the open-ended approach to encourage participants to explore emerging themes of interest more freely, thereby opening the potential for previously unexplored ideas to be investigated [101]. Common themes may typically derive from richer, more in-depth data sets and leads to the development of theories and/or detailed conceptualisations of categories of interest. We used the inductive approach of moving from specific interviews to common insights and patterns of meaning, allowing the data to drive the research [96,100]. We checked for researcher bias using techniques such as personal reflexivity and conversations among the research team [102,103].

### 2.1. Interview Participants

We approached prospective participants via their publicly available email addresses. Participants were selected for their varied expertise and/or experience concerning human–animal relationships from a wide range of relevant contexts. Participants needed to have close experience with animals or proficiency with animal topics via their work and/or other experience with animals. Closeness here is defined as a relationship with other animals that at least requires significant animal/species knowledge, expertise, and experience, as well as an understanding of human–animal relationships, including but not exclusive to people who feel personal closeness, respect, and/or empathy for other animals as demonstrated via their advocacy work, academic publication, or authorship. The selection criteria that participants were required to have are as follows:

- worked or are working with living animals in a range of contexts, and/or
- contributed to critical or deep thinking concerning human–animal relationships, and/or
- represented animals in public discourse.

Most of our participants were selected for their academic/research expertise or other relevant skills in various types of animal work. These fields included animals in political science (*n* = 1), psychology and philosophy (*n* = 2); biomedical and toxicology research (*n* = 2); animal welfare science, policy, and law (*n* = 3); wild animal conservation, ecology, and management (*n* = 3); sociology, media, and literature (*n* = 2); political representation (*n* = 1); and animal farming (*n* = 1). Some of these participants had additional significant advocacy expertise or experience related to improving or reporting on the lives of animals in industrialised animal agriculture. The participants came from different countries, including Australia, Canada, USA, Portugal, Germany, Finland, and the UK.

### 2.2. Interview Procedure

We received human ethics research approval (ID SEBE-2021-MOD02) for this study. The question guide was developed by the researchers based on initial readings and discussions surrounding voice and speaking for animals (see Supplementary File S1 for the question guide). Author AT conducted the interviews between February and May 2022 online via Zoom video and/or phone calls (per participant preference). Each interview lasted 30–60 min and, with written consent, was recorded.

The interview included questions concerning how people speak for animals, the participant’s thoughts about what it means to reliably represent animals’ interests, how animal representation could/should manifest, and what relationship with animals and personal characteristics someone would need to be able to reliably speak for them. We added a personal dimension by asking participants if they had individual experiences with speaking for, or interactions with, animals and, if so, what their thoughts were related to these experiences. The interview schedule was loosely adhered to [97] (pp. 62–82) as the participants often initiated their own elaborations, and as such, needed no additional prompts from the interviewer in terms of keeping the responses related to the questions. At the end of each interview, demographic data were collected related to age, gender identity, and rural/urban location for aggregative reporting. The participants were given the choice of creating their own pseudonym or having us create one for them. The interviews were transcribed intelligent verbatim (omitting unnecessary or filler words) as the key focus was to extract meaning. The participants were not offered incentives or other rewards for their contributions. Themes and concepts related to speaking for animals were explored until it appeared that no new relevant substantive insights and themes were being introduced by participants, which resulted in a total of 15 interviews.

### 2.3. Data Analysis

Author AT analysed the content of each interview inductively, guided via the methodologies of reflexive Thematic Analysis (Big Q Qualitative Analysis) [98,104]. In qualitative analysis, data collection, memoing (descriptive/interpretive notes), data analysis, and coding and theory development often occurs simultaneously, guided via a “constant comparison analysis” which refers to the method of comparing emerging findings and meanings with existing findings [105,106,107]. The preparation included reading each of the transcripts a minimum of three times to become familiar with the content of the individual interviews. Author AT used a methodological journal and conceptual mind mapping software to reflexively record memos, patterns, themes, and sub-themes, as well as their potential relationships and/or dissimilarities. The data from the initial interviews were used to identify major themes to be used as a guide for subsequent theoretical sampling (i.e., selecting future interview participants based on their ability to elucidate and expand their understandings and insights of emerging theoretical domains). Themes were compared with the literature from a combination of critical animal studies and other related humanities disciplines. At the coding level, the interpretation was partially driven via semantic (participant-driven, explicit meaning) and latent (researcher-driven, conceptual, implicit meaning) levels [98]. We used selected quotes to illustrate aspects of the subthemes.

## 3. Results

Participants provided varied and rich perspectives based on a wide range of experiences and relationships with animals, including wild animals, so-called “pest” animals, domestic animals including animals in biomedical research, companion animals, and animals in the entertainment/racing industry. However, despite the various background of the participants, farm animals were commonly referred to as examples of the concepts they were describing.

The reason why animals in production tend to be a common discussion point is: (1) the sheer number of animals affected, and (2) the profound social and environmental/ecological/climate impacts of factory farming [26,108]. They are furthermore a common and obvious example of human overreach. A significant portion (62 per cent) of the Earth’s total biomass of mammals are trapped within human industrial systems [109], and as outlined in the introduction, industrial practices significantly impact ecological systems and the animals that are part of those. The situation for domestic animals and wild animals may therefore be considered aspects of the same issue [31].

Participants provided varied and rich perspectives based on a wide range of experiences and relationships with animals, both wild and domestic. The major theme was “Animal voice”, which represents the participants’ perceptions of what *animal voice* is, and how human language constructs and paradigms shape the realities of other animals (Table 1). Participants identified several important properties that constitute this theme. Below, we explore the theme of “Animal Voice”, its subthemes, and its properties within the context of ongoing discussions in the literature.

### 3.1. The Animal Voice and the Role of Human Language in Shaping Perceptions

The theme describes participants’ experiences and reflections on common perceptions related to the concept of *animal voice* and how the human language can be used to construct stories and paradigms which may prevent us from thinking about animals as being able to communicate. The theme describes how current anthropocentric paradigms influence how we approach scientific research on animal communication and how conceptions and messages about animals as voiceless may perpetuate anthropocentrism, even in animal advocacy. While participants emphasised that scientific theory and research, as well as experience with animals are crucial aspects of understanding, a complete understanding of animals requires that we challenge and attempt to move beyond current scientific definitions and paradigms by working for and with animals.

Most participants explicitly or implicitly stated that animals have a voice and that they are communicating; that animals will speak for themselves if we dedicate attention and time to listening, observing, and understanding. They highlighted the importance of experience with animals through intimacy (active listening, patient observation, and interaction), reciprocity, respect, and compassion. This includes acknowledging animals as individuals and agents of their own lives and experiences, as well as recognising their individual wants and wills. This implies that we must begin to seriously consider and include novel and alternative methods to understand animals beyond the limits of biological/physiological needs and behaviour.

Participants furthermore touched on complementary ways of understanding nonhuman animals, drawing analogies with certain groups of similarly vulnerable humans. The subthemes will be discussed in more detail in the sections below.

#### 3.1.1. Animals Do/Do Not Have a Voice

*Animals already speak/communicate*. Some participants highlighted that speaking for animals is a modern and predominantly Western problematic concept because it perpetuates misguided and/or inaccurate human constructs about *animal voice* and communication, effectively disabling the agency and voices of animals:

“*I think within this term, speaking for animals, that’s the danger and tendency, that what you’re doing is actually replicating the idea that animals cannot communicate and aren’t able to partake in co-creation of space or ideas or community. For me, the concept of speaking for animals is within this Western, White, European enlightenment, colonial mindset. You don’t get it in Indigenous mindsets. Animals speak for themselves there. They absolutely speak for themselves through spirit, through mystery, through myth…*”

(Mischa)

Other participants explained that we perceive animals as voiceless because our current understanding of voice and communication tends to be limited. Our perceptions and depictions of animals as voiceless are artificial constructs, but have become normalised because we are conditioned to limit listening and understanding to verbal communication:

“*…There’s a fantastic organisation in Australia called Voiceless but I continue to disagree with the name of their organisation because they (animals) do have a voice and they do communicate—we just don’t share a common language….and so it is common to say they’re voiceless because they don’t speak our tongue…*”

(Snowy Owl)

Several participants expressed their reluctance to speak for animals. Years of observation and experience had left them with a great sense of awe, admiration, and respect for the animals, and as such, did not feel it was their place to speak for them:

“*I think it’s highly arrogant of me to think I can speak for a snake. I’m just not comfortable with that…I just feel that it would be limiting of me to try and do that because the snake is so much richer and more sophisticated than I will ever be.*”

(Snake Advocate)

One participant framed *animal voice* in terms of consent and new approaches to listening. To determine whether our actions for animals are foregrounding their interests and needs, we must begin by paying attention to their various means of communication. Since we are interacting with or observing individuals who communicate differently, the first step is to approach listening differently:

“*I think the issue of consent brings an important issue to the fore of, is this a self-proclaimed right to decide what is best for animals from a human point of view? The first step there to minimise that would be to listen to animals, and listening would be more than theoretical, through for example science and seeing how they behave, what their ethology is. Listening is a physical act as well, and it involves proximity, it involves sharing the landscape.*”

(Cat Man)

The issue of animal consent and agency was also discussed in terms of personal and organisational approaches to speaking for animals, and how these approaches are shaped by cultural norms about the subordination of animals—that we may assume a right to act on behalf of, or speak for, animals:

“*…and I get that some animal advocates say that’s at the heart of the subordination of animals that we just feel that we can, with very limited authority or knowledge interfere in the lives of animals who don’t understand as well. And talking to someone who’s working with an animal psychologist recently it actually came home to me how I don’t know anything about how dogs socialise—and still unreflexibly willing to intervene in an animal’s activities on that basis. And then we have people who work for advocacy organisations. And some of those people seem extremely knowledgeable about animals, and some of those people seem extremely unknowledgeable about animals—but by and large, many of them are to some degree self-appointed individuals.*”

(White Ibis)

*Sometimes animals need us to speak for them: contextual voicelessness*. Some participants emphasised that sometimes humans need to speak for animals because there are contexts where animal voices are not being heard or ignored. For example, increasing urbanisation and the covert practices of factory farm animal industries have disabled animal voices through their enforced separation from humans. Many people have lost the opportunities to meet, become familiar with, and become acquainted with animals intimately, thereby also removing farm animals’ opportunity to be seen and heard by people:

“*They desperately need us to speak for them…I mean animals on the one hand, have no option. They have no voice as it were. They can’t speak for themselves as it were…I have sheep here, rescue sheep. They do speak for themselves. But the way they speak takes a lot of experience to hear anything that they are trying to say…. So I would say, ideally, people speaking for animals have experience of animals. But 95 per cent of Australians live in cities. They don’t have that kind of opportunity.*”

(Daniel)

One participant evoked the experiences of animals in a human-dominant world by drawing the analogy with vulnerable human children who may be unable to speak for themselves, defend themselves, or represent their own case in a world made for adults:

“*So, I think what we need is to change the paradigm. We have to think about animals as we think about our children, vulnerable creatures that we need to protect, look after and you only stress or harm them when it is very important for their own good. I only subject my dog to a vaccine if it’s for their own good*”

(Maria)

Similarly, another participant described *contextual voicelessness* as the silencing of animal voices through the structural processes of stigmatisation in policy and legislation, drawing parallels to stigmatised groups of humans:

“*Animals to me are the most stigmatised and silenced social group, like to me, they are a social population in the same way that we have different human social groups that are stigmatised and silenced—animals don’t get to have a voice at all and that’s existed for other human groups over time and the way those groups are silenced is through structural stigmatisation through language, in particular policy and legislation. So, the language that we use to talk about those issues and those beings is designed to silence them or make them invisible or undermine their right to have a voice—or the meaningfulness of their voice.*”

(Gunter Goose)

Participants discussed aspects of animals’ *contextual voicelessness* that is perpetuated through our food choices. As long as we let our perceptions and attitudes regarding animals as food continue to dominate our actions and thoughts, we are also willingly closing our ears to animal voices. Some suggested that our obsession with meat means most of us will not be open to considering animals as having voices because hearing their voices may be too confronting.

“*So listening to animals is […] less than a priority, it’s like negative priority […] we want the opposite of listening to animals—we don’t want to hear them at all. I mean, we’re breeding meat so that we can still eat meat and […] we’re doing cell-ag, which I think is a good thing and hopefully that will help change the world but, we want meat so badly that we’re investing billions every year so we can grow it in a lab so that it doesn’t have a brain it just has flesh so that we can continue on eating this thing that we really want—without having to deal with the individual—and any of the (laughs) communications that would arise…*”

(Snowy Owl)

We may be happy to listen to people who speak for the animals we are close to as companions but feel uncomfortable when being confronted with the prospect of similarly considering the more distant animals we want to eat or those we consider inconvenient.

“*…the general public as animal using community does express a concern for the wellbeing of all animals—but we know that companion animals are treated different to production animals and animals that are kind of classified as pest animals—and there were some interesting studies that were done around cognitive dissonance that shows that people do not really want to talk about animals that they consume… particularly the closer they get to a consumption […] so, I guess in that kind of ‘who gets to speak for animals’ […] is also who is listening.*”

(White Ibis)

*Knowledge deficiency of animal language and communication*. Participants emphasised the importance of recognising the growing science on animal communication, language, and behaviour, especially those that document the emotional/subjective experiences of animals that we do not necessarily (want to) see or hear:

“*…he tells me stuff that he has seen and learned in labs, like for example—the vocalisations of rats—this is about their communication and our listening and discerning and interpreting—and I didn’t know until he told me about rats’ laugh […] they have high pitched sounds of joy when they’re with their loved ones—and if they’re having a good laugh, like if you’re tickling them—they’ll have a really good laugh […] and the study has shown that this is happening, which makes it all the sadder that we can’t hear that…*”

(Snowy Owl)

From the perspective of someone with many years of experience with animal behaviour and wildlife management, one participant heralded significant future changes by pointing to emerging scientific trends. We are only just beginning to scratch the surface in terms of understanding the many different ways that animals communicate:

“*Nature does not usually allow for frivolity, birds sing and communicate information for purposes that we think simplistically advances their reproductive success or their dominance over other birds—but there’s a lot more going on than that, and I think we are just beginning this current phase of understanding animal behaviour…*”

(Old Man Winter)

#### 3.1.2. Human Language Creates Realities and Paradigms

*Misguided conceptions and messages; inconsistency, speciesism, anthropocentrism, and othering.* Participants described our historical use of language to describe animals and the socio-psychological factors, including human superiority and dominance narratives that have become so ingrained in our culture, language, behaviour, and actions that we no longer question them. Via these narratives, we have become conditioned to minimise animal experiences and maintain their separation from us by ignoring their voices:

“*…I think because we don’t have a history of paying close attention, it’s a history of othering—of diminishing their value and their worth in the hierarchy we’ve created about humanity, all of these reasons for us to not listen to animals, not to pay attention…*”

(Snowy Owl)

Some described the theme in terms of anthropocentrism and the social exclusion of certain animals (speciesism):

“*In fact, I consider this all the same fight pretty much and the same challenge, the challenge being anthropocentrism as a paradigm, as a worldview. If you look at it that way, then we’re talking about folks who I think can speak for animals are folks who treat this not as an environmental issue, not as an ecological issue but as a social justice issue, in the same way that you would treat any environmental justice issue to more vulnerable marginalised human communities.*”

(Cat Man)

One participant described how we can change paradigms with relative ease to either benefit or exclude animals. Institutional re-structuring of relationships via the arbitrary categorisation of certain animals may dictate how we speak for them or how we choose to frame debates related to them. While these changing relationships may not affect us, they may have profound effects on animals and how we view or treat them.

“*…so thinking about the Royal Society for the Protection of Animals in Queensland it was originally the Royal Society for the Protection of Animals and Children ….and so, we see these things as separate today but once upon a time they were actually much more conjoined—I think it points to that we have become calcified into a particular form of structural relationship around the debates about animals—and that’s not necessarily a natural or necessary thing—it could be subject to contestation.*”

(White Ibis)

They also provided an example of how institutional categorisations may shape human compassion and moral concern for certain animals, and therefore, how we choose to speak for them, depending on the geographical context. In landscapes where ecological communities are largely changed by humans, certain animals have become particularly vulnerable as they are judged according to what we currently value as aesthetically important to that particular landscape:

“*…every country has its own taxonomy of animals and what animals are—in Australia the possum is a wonderfully cute animal that you would create an effigy of to give to a child whereas in New Zealand, the possum is an invasive species, and you might recruit children to systematically cull them at the schools…and […] particularly in regards to animals, there’s no rhyme nor reason to the inclusion or exclusion of different animals from the circle of compassion and that’s the way in which people will speak for them.*”

(White Ibis)

One participant described how *inconsistent attitudes* to different animals means certain animals are rejected as worthy. We stigmatise some animals as undesirable or unwanted according to inconsistent rules. As such, some animals may be perceived as undeserving of representation while other animals are represented because they fit in with the preferences of certain groups of humans.

“*I don’t think that you can tailor your speech for animals by saying these are the animals we can speak for and these animals we can’t. I don’t think you can pick and choose because then you’re imposing an aesthetic on the world. You know, it’s an aesthetic. It’s not consistent.*”

(Daniel)

Institutional inconsistency in attitudes to animals is similarly reflected in the way humans design animal law, which may arbitrarily protect or not protect animals depending on the context in which animals are placed by humans. Animals are often not given a choice in where they are placed, with whom, or how they will be perceived according to the context. When we perceive animals as having no agency or voice, we assume a right to change their legal status according to which anthropocentric values are relative to the context: economy, hedonism, aesthetics, convenience, or similar utilitarian justification to favour human interests:

“*In Victoria, we have codes of practice for different species of animals. Only the ones that relate to cats and dogs are mandatory to follow and illegal to break, the ones for agricultural animals and racing animals, they are all just voluntary, so it really doesn’t give them any sort of protection…a greyhound will be in the racing industry and basically have no protection and then as soon as they finish racing and get adopted as a pet, they suddenly have some sort of legal protection when they’re the same animal—their situation has just changed so, it’s a very clear issue in terms of how we view them*”

(Gigi)

Similarly, while constitutional changes or changes in animal law are important steps forward, these may be more symbolic than reflective of immediate or authentic intention to implement meaningful change for animals.

“*Legally, it doesn’t have a strong direct impact on actual substantive duties towards animals under the law. It depends on how sentience is recognised and whether it is attached to actual operative provisions within the act. But at the moment, it’s just more symbolic.*”

(Shy Albatross)

While several countries now legally recognise animals as sentient, limitations render the recognition with limited operational power as long as the more powerful voices of the animal industries are prioritised at governmental decision levels:

“*…I think that is subject to contestation, but I think that’s really interesting how theoretically that is supposed to be a game-changer […]—but yet there is massive continuity, and it just shows how power is really written into the fundamental systems of production that are resistant to change—*”

(White Ibis)

While the official recognition of sentience may be perceived as important at higher levels, members of the public react with puzzlement to the idea that sentience in animals needs to be recognised by the law:

“*So yeah, I think it’s positive. It’s funny because we campaigned to have sentience recognised in law. Then when people ask you like, well what does that mean, and you say well, it’s just about recognising the fact that animals can feel fundamentally. People sort of look at you like you know, who denies that?*”

(Shy Albatross)

*Scientific reductionism, ontological, and epistemological approaches to understanding animal voice*. Participants explained that we have made assumptions about animal communication through an over-emphasis on *scientific reductionism*. As such, participants explained that human willingness to consider *ontological and epistemological alternatives* is the key to understanding *animal voice*. Animal language and communication exist regardless of how humans define them or choose to interpret them—we have, until recently, not focussed on different approaches to understand animals beyond biology, physiology, and behaviour. Our claim to speak for animals is limited if we do not understand animals as individuals with their own unique wants and interests.

“*…We’re conditioned to listen for a language whereas animals are often quite embodied. You don’t just listen with your ears, or you can’t say you’re listening to animals because you read twenty papers on the ethology of these animals and suddenly, you’re an expert on them—your credentials speak for them almost—when you don’t know them…*”

(Cat Man)

Participants also highlighted how scientific reductionism manifests as a tendency to present one-dimensional images of animal lives and experiences. Rarely do we consider speaking for or representing animals in terms of personality and subjective experiences:

“*We’re judging on something which, you know, the focus is very narrow. It’s all about harm, it’s suffering, it’s pain and it’s that what we are talking about when talking for the animal. Mostly what we are not doing is about the daily life of an animal. It’s not the feelings of the animal. It’s just does it like to talk to you, does it want to look at television, does it tell you to put on the radio or whatever? So, there is more than this actually, but we are very narrow in the discussion. It’s not the whole total behaviour and living of the animal we are talking about when we talk about speaking for an animal.*”

(Junior)

Using scientific reductionism, we have created a mechanistic framework from within which exploitative industries can more easily justify their instrumentalisation of animals, rendering animals vulnerable to harm, misrepresentation, and the suppression of their voices.

“*I think it leads on from what we were just talking about in terms of the physical scientists and the reductive nature of positivist scientific discourses. So that the fundamental underpinnings of that kind of science can lead you down very blinkered paths that don’t take into consideration the feelings or thoughts of animals in a meaningful way. It’s only about observable traits and responses. That’s a dangerous thing when you see agriculture industrialising more and more. Animals being put in sheds and vets being tasked with keeping them alive*”

(Busy Bee)

Participants also described the issue in terms of limited ethical, legal, and welfare science knowledge within the agricultural animal industry:

“*…there are a lot of vets who don’t have that [legitimacy to talk about animal welfare science, ethics, and law] and are still considered and taken by government and industry to be experts on animal welfare which I think is really interesting because in reality the animal welfare that’s taught in vet schools is tiny…we know more about wildlife than animal welfare in terms of what we’re taught, and we don’t know much about wildlife…*”

(Blue Angel)

Participants explained that *understanding animals* may also include coming to terms with our own limitations of understanding—a different and perhaps more profound sense of appreciation of animals that is not defined by or limited to scientific knowledge. One participant illustrated this concept as contemplating the prospect of other animals’ existence throughout deep time and comparing with the relatively limited time that humans have existed. This idea challenges us to situate humans into a different temporal perspective in which we are no more than animals to have perhaps relatively briefly existed among a long line of other animals. From the perspective of deep time, mystery, and a sense of humility, we may come to appreciate that animal lives are and have always been as rich and eventful as ours—regardless of our existence and our understanding of them.

“*There are guestimates, there’s great science on evolutionary biology, but they’ve [snakes] been here a long time and I just think there’s so much that we don’t understand about these animals, and I love that. I love the fact that we’ll never fully understand how they navigate, how they feel, how they communicate with each other. That’s why I feel like I’m maybe not your orthodox scientist who wants to know everything.*”

(Snake Advocate)

#### 3.1.3. Let Animals Speak

*Communication is more than spoken language.* Some participants explored the concept of *animal voice* in the context of companion animal relationships and the importance of *listening* to animals. Key words included openness, observation, reciprocity, closeness, respect, and intimacy. While many of these relationships may typically be characterised by human dominance, some also provide powerful examples of respectful and reciprocal communication and intimacy between species. We need to recognise these relationships culturally due to their profound nature:

“*Listening to the movement, sounds, demands and needs of the domestic companion in the domestic space is really important. The more you do that, the more you deepen your relationship. I think it’s much more profound. I think a lot more people know it and it would be great if we had more cultural recognition of how profound that is.*”

(Mischa)

Similarly, another participant described how their first experience of living with companion/domestic animals (a cat, two dogs, and several chickens) profoundly changed their perspective in terms of understanding animals. Coming from a scientific background, the experience made them see animals from a different perspective; as individuals who each required very specific approaches depending on their personal needs, wants, and moods:

“*They [animals] have been the primary teachers of—are we actually listening to what these individuals have to say? The only way I would have ever been able to figure him [cat companion] out is just spending time next to him, not only observing him and complying with his cues but—just observe what this individual wants, what he finds pleasurable when I am not engaging with him as well… It speaks so much to the physical aspect of listening…that the individuals need to get physically comfortable with you before they start showing you who they are…I thought that a cat is a cat, and a dog is a dog*”

(Cat Man)

*Amplify animal voices*. One participant described representation in terms of ambassadorship and familiarity with wild animals via education and exposure. Via their work as a highly skilled, knowledgeable, and passionate educator, their role expanded to become one of both advocate and diplomat. By allowing people to experience a different perspective of snakes through the compassionate lens of the educator, the animals commonly perceived as hostile or repulsive are presented in a light to reveal other dimensions of who they are. Animals are thus transformed from being perceived as repulsive and/or a potential threat to humans, to becoming individual beings with their own important lives, wills, wants, and sense of place in the world.

“*I can take all my insights and experiences and try and bundle them up into something that people think, oh, next time I see a snake, I’m not going to freak out. I’m not going to use that shovel. I might just leave it alone.*”

(Snake Advocate)

Rather than speaking for animals, advocates should strive to act as amplifiers for their voices and interests:

“*It’s the paradigmatic structures of anthropocentrism and human exceptionalism that need to be dismantled, and we can’t dismantle them by doing piecemeal changes through anthropocentric practices. That is not going dismantle the overall structure. I think this whole idea of speaking for the animals because they can’t speak for themselves is that anthropocentric reinforcement, whereas speaking for the animals to amplify their voice and advocate for them in ways that they don’t have access to, is at least attempting and non-anthropocentric practice.*”

(Mischa)

Participants furthermore discussed alternative ways that humans give animal voices a platform, such as via various art media or cultural community:

“*I think it’s absolutely fine to experiment and explore representing animal language in human language, with understanding the caveat that we can’t ever do that properly…I think that is actually a really interesting area for exploration. I think all of those [creative writers], or certainly Gene Stone, certainly Laura Jean McKay, they would consider themselves advocates for animals. They are performing it outside of standard advocacy space, but they are advocating for animal representation. I don’t have an answer or theory about it, but I think it’s a really valuable space for exploring this question…*”

(Mischa)

Others described the potential for tapping into a public thirst for learning about animals and emphasised the significant influence through which various forms of cultural expressions, such as games, can bring animals to our attention:

“*… and this game, Wingspan, which is produced by female game designers, was hugely successful and sort of talks about this great cultural thirst for animal related stuff in every kind of area, and in the sense the game itself is about animals and laying eggs and their lifecycle […] that represent talking about animals and learning about animals in different ways… I just think on a kind of practical everyday basis it really is people within the cultural industry who predominantly dominate the talking for or representing animals—not really advocates and activists*”

(White Ibis)

## 4. Discussion

We interviewed 15 people with deep, sustained, and diverse experience and knowledge of animals to gather insights about current thoughts and perceptions related to animal representation. We used an inductive, qualitative approach to develop new ideas and to deepen the understanding of current and emerging concepts. The major theme, *animal voice*, represented common topics in the interviews and the participants elucidated several subthemes and their properties. Participants described *animal voice* in the sense that *animal voice* is currently contextually defined and/or limited by humans. This renders animals artificially voiceless and thus vulnerable to misrepresentation and harm. The subthemes of *animal voice* represent different aspects of how we may assume to speak for animals or seek to empower animal voices based on different conceptions and constructs related to *animal voice*.

**Animals do/do not have a voice.** The issue of misrepresentation of *animal voice* was evident in the participants’ reaction to the implicit suggestion that speaking for animals is necessary because animals cannot speak. As one participant summarised, animals will speak if we are willing to listen and observe. Some explained that the term “voiceless” risks perpetuating the anthropocentric idea that animals cannot speak or communicate. It may additionally perpetuate the idea that animals have no agency, suggesting that humans therefore need to interfere and take charge of their lives.

Other participants expressed a reluctance to speak for animals because of their profound sense of humility and respect for them. They described their own as well as other alternative methods to convey the plight of animals. This included providing animals with a communication platform via various media and the arts or via education. For example, they described how sharing discovery and exploration through the lens of someone who profoundly respects and cares for animals presents people with the option of learning through compassion. At the same time participants were clear that compassionate approaches need not exclude traditional scientific knowledge, but rather that the added dimension of compassion allows us to discover animal voices in ways that have been understood mostly through intimate human/animal relationships. Learning can thus expand beyond the limited scope of just satisfying our own curiosity or as a means of fulfilling the criteria for certain institutional learning processes. It may elevate learning to recognise and nurture our own potential compassion for animals, which can be a profoundly rewarding, insightful, and healing personal journey [110], both for ourselves and the animals we extend our compassion towards.

Some participants provided a different perspective on *animal voice* and discussed how voicelessness is imposed on animals by various oppressive institutionalised systems. There were parallels drawn with vulnerable humans such as children, who may be considered voiceless because they depend on trusted others to speak for them in a world of social, political, educational, and economic systems designed by and for adults. In the industrial/institutional context, animal voices are relevant only (if at all) in terms of being instrumental to aspects of the scientific and operational processes for optimising production [111]. Acknowledging agricultural animal voices thus begins with acknowledging the reality which has rendered them contextually voiceless—the commodification of them as “meat” (discussed in more detail below).

*Knowledge deficiency of animal language and communication.* Some participants explained that our concept of *animal voice* and communication may be limited because human constructs of language are narrow. Participants described how we are only just beginning to take an interest in this aspect of animal lives. As an example, they discussed the importance of recognising the unique and varied approaches for the shared understanding between humans and animals in intimate relationships. As noted in the introduction, similar ideas have long been discussed in the literature. In 1982, Levinson called for the developing field of human–animal relationships to become a discipline in its own right, emphasising the importance of recognising both intuitive and scientific methods as equally important aspects of understanding animals and their communication [112]. Until recently, this literature has largely focussed on companion animal relationships from the perspective of human benefit. This instrumentalisation of companion animals has ignored the animal perspective and the possibility that these relationships may be more dynamic, reciprocal, and profound than previously assumed. Over the last decade, this recognition has prompted an increase in studies that have explored companion animals’ perceptions and reactions in human environments [113].

As participants noted, communication in other animals is often nonverbal. It can be embodied, chemical, and/or visual, and also manifests in various patterns such as in the song of birds and whales [114,115,116]. Recently, machine learning to decode animal communication has become more widespread [117], as demonstrated in attempts to find ways to decode the languages of sperm whales [118] and song birds [119], rather than just recording and describing their various species manifestations and patterns. The Earth Species Project is in the process of applying machine learning to understand communication in primates and some species of birds. Fitch (2019) has furthermore argued that human conceptions of language need to expand beyond communication because we are missing an important aspect of understanding in animals. Fitch (2019) stated that the fact that some animals do not express understanding does not mean they do not possess understanding [120], as documented by a large body of the literature on animal cognition [121]. The concept of animal understanding is being similarly explored by artists and researchers at the Interspecies Communication Research Initiative (ISCRI). This research aims to address questions of what nonhuman intelligence is and where in the body it is located, involving developing novel approaches to gain insights about the internal lives of octopuses via the close study of AI/octopus interaction.

As reflected by our data, scientific research and general attitudes are as such gradually changing how we approach the understanding of animals, and importantly, shifting the focus towards capturing animal understanding and perspectives. However, some participants reflected on the extent to which we can assume a right to understand animal voices and, if so, how do we determine the best ethical approaches to advance this understanding? These issues have been discussed in depth by Eva Meijer in their groundbreaking book *When Animals Speak* [122] and with Bovenkerk (2021) in “Taking animal perspectives into account in animal ethics” [67]. While critical to anthropocentric approaches, Meijer and Bovenkerk argue that to be able to make democratic decisions with animals, we need to recognise animal agency as an essential first step. Meijer and Bovenkerk furthermore argue that we need to develop better relationships with animals by improving our understanding of their differences in expression, wants, and wills [67] (p. 50). This can be achieved using alternative methods that do not reinforce the anthropocentric oppression of animal agency. Meijer (2019) suggested one approach is through experimental settings in sanctuaries or multispecies households where the animals themselves can determine the grounds for interaction [122] (p. 239).

In terms of institutional perceptions of *animal voice* and agency, the constitutional recognition of animals as sentient beings and existing laws to protect animals in some countries [123,124,125] may reflect a positive institutional paradigm change in human attitudes to animals. However, as some participants emphasised, even constitutional sentience recognition continues to have little meaningful effect as long as there are legal inconsistencies, codes of practice, and provisions that enable economic/legal [88,108,126], aesthetic [127], nationalistic/cultural [128], ideological/supremacist [129,130,131], speciesist [132], and hedonistic [133,134] justifications to suppress animal voices. Even if animals are to be constitutionally included similarly to humans, the challenge remains that our current democratic and judicial processes rest on the assumption that constitutional actors have the ability to assert their own interests [123]. Eisen (2018) has argued that for this reason, we need to consider expanding the discourse on constitutional animal protection to include the consideration and protection of subjects that are unable to or unwilling to “speak in the language of the law with their own voices.” (p. 909).

**Human language constructs realities and paradigms.** The issue was discussed in terms of how the old paradigms of human superiority and dominance perpetuate anthropocentric thinking in many human systems, but that these attitudes are gradually changing. Humans are increasingly questioning past assumptions of sharp divides between humans and nonhumans, and with this acknowledgement, some biologists are even beginning to challenge the concept of sharply divided species boundaries (see [135,136]). The scientific and philosophical discourse has thus increasingly focussed on arguments that basic emotion and social intelligence are not just fundamental in human animals, but also in other vertebrate and many invertebrate nonhuman species [31,43,125,137,138,139,140,141,142,143,144,145].

Participants drew parallels between the traditional ways in which we have perceived groups of marginalised humans and how we similarly view animals. Rather than viewing certain traits and abilities as exceptional to humans, we are beginning to recognise that these and other abilities are present in most sentient, if not all animals, and that species differences perhaps ought to be viewed on a spectrum rather than by rank [135].

Issues regarding mental and physical ability have been similarly discussed in terms of how human language shapes how we think about and treat people who fall outside able-bodied norms [146]. For example, the UK-based Union of Physically Impaired Against Segregation described the word “disability” (a negating term) as a form of oppression that only adds further to the burden of their already existing challenges [147]. Similarly, the words “voiceless” and “animal” may contribute to perpetuating biased negative perceptions of animals as they conceptually separate and categorise animals negatively against humans. Sunaura Taylor (2017) drew this parallel explicitly in their book *Beasts of Burden*, positing that mental and physical ability is not what gives living beings dignity and value. As with humans with different abilities, we have constructed a story of human dominance over animals by comparing their abilities and traits to our own [146].

Meijer (2013) has similarly argued that the hyperfocus on discourses regarding animal emotionality, consciousness, and cognitive and physical ability has distracted from a fundamental ethical argument that the social and political inclusion of nonhuman animals need not be contingent on their intrinsic capacities and/or human perceptions of moral agency. Abbate (2020) furthermore suggested an important first step would be to consider that animals, like humans, have intrinsic worth, independent of their value to humans. This means we need to respect animals in terms of how we treat them, but also how we talk about them [148].

*Scientific reductionism, ontological, and epistemological approaches.* Moreover, participants discussed how animals are deprived of their voices via an over-emphasis on institutionalised objective, positivist, and deceptive language in animal science and welfare policy and the linguistic mechanisms to discourage the humanising of animals in industry/educational institutions. These institutionalised mechanisms have also been brought to attention by authors who describe how non-emotive terms may be deliberately or unconsciously used to desensitise us to the reality of animals [149,150] or to facilitate dissociation by discouraging the use of humanising terms to describe animals [151,152,153].

Other authors have discussed how these mechanisms are also prevalent in our daily language. For example, Adams (2016) explained how we create “false mass terms” by wrongly lumping individuals under certain umbrella terms. The mass term “meat” is used to define entire species, transforming animals from sentient individuals to “food” (another mass term), protecting us from the discomfort of contemplating the remnants on our plate as those belonging to a destroyed individual [152]. Adams (2016) explained that via the use of dissociating terms, we grant ourselves perceived license to eat animals as it is no longer considered contact with the animal, but contact with “food” [152] (p. 6).

Some participants similarly reflected on the many ways in which humans deal with the personal discomfort (“cognitive dissonance”) that arises as the result of wanting to eat meat, while at the same time having awareness of the confronting realities of factory farm animals. In the literature, this has been coined as the “meat paradox”: while we are concerned for farm animals, we simultaneously continue to find ways to suppress the underlying discomfort in contemplating the lives and deaths of the animals we wish to keep consuming [154,155].

Participants discussed other issues with the over-emphasis on reductionistic thinking and how this paradigm is being increasingly challenged by the billions of humans globally who regard their companion animals as full-blown family members with their own meaningful and rich experiences of the world [156,157,158]. While the power structures in these human–companion animal relationships still require closer examination [159], many transcend species barriers as evident not just by the strong emotional bonds that are developed between humans and nonhumans, but also the reciprocal nature of interaction and the development of common understanding [112,160]. By including animals into our moral circle, we are also more likely to extend prosocial behaviours toward them [158], giving their voice validation.

This concept was vividly illustrated by participants who described their own eye-opening experiences of sharing a home with companion animals. It was also described by those who had devoted their lives to the close scientific study and/or advocacy of wild animal species, those who had tirelessly devoted their lives to advocacy work for farm animals, or those who had spent a significant part of their lives volunteering in rescue animal shelters. Their accounts documented that intimacy with animals is a crucial aspect of listening to their voices and thus requires an open mind as well as a respectful, compassionate, and patient approach, similarly to how we would interact with other humans.

Some participants noted that it may be argued by some who are closely involved with animals that their close proximity to and experience with animals implies they are their best care providers. However, physical proximity does not guarantee or promote best care practices [161] and may indeed tell us very little about the true nature of the human/animal relationship/connection.

This concept was illustrated by Gruen and Jones (2015), who drew critical attention to the “romanticisation” of the “connection” that people who are uncomfortable with factory farming (“compassionate carnivores”) imagine with the animals they kill and consume. Somehow, being involved in every step of the process of slaughter is viewed as a more ethical form of consuming animals, as killing the animal one has raised is thought to increase a sense of humility and respect for the animal. And, while this may be true to some extent, the authors argue this connection may not be as genuine as much as a false connection [162]. The authors refer to Katie Gillespie’s (2011) concept of “connected disconnection” in which the process of killing necessitates emotional distance from the animal, which is made possible by connecting to the violence against the animal [163] (p. 120).

Whether we are involved directly in the killing or paying someone else to kill, Griffin and Griffin (2021) argued that because animals are social beings and part of our social world, our violence towards animals is a social harm affecting not only animals, but also humans and the environment [108]. These harms include but are not limited to the documented harms associated with slaughterhouse work and meat consumption, but also harms that correspond closely with what Adams (1990) identified as the underlying sexist and patriarchal devaluation and feminisation of compassion for animals and nature [164,165]. Brooks Pribac (2016) furthermore noted how caring for animals is invalidated as a form of weakness or even seen as pathological—and how the associated systemic processes of dissociation not only contribute to obscuring animal suffering but may also lead to the dismissal of those who advocate for them as over-emotional, or it may even lead to the victimisation of the “perpetrators” (farmers and consumers) (p. 56). The author describes these processes as a betrayal not just of the animals, but also those who are unwittingly part of the supporting mechanisms of their exploitation [166].

Abbate (2020) has described the social pressures that contribute to drive meat consumption despite people’s awareness of factory farm animal suffering. Aspects of “social duress” may include various socio-cultural environmental factors that pressure individuals to comply with contextual meat-eating norms and traditions for social acceptance. Abbate (2020) thus noted that while consumers of factory-farmed meat can be argued to be partially morally responsible for the violence towards animals, it is important to consider that they may be acting under social duress which causes *volitional impairment*: “One need not have a gun pointed towards one’s head to act under duress” [167] (p. 11). Abbate’s concept of social duress and volitional impairment could be expanded to include ideological, political, and academic domains where moral and ethical discourses on animals may affect individuals who try to speak for animals, and therefore, also influences where the discourse is headed.

The connection and closeness with animals identified by participants was described as profound, reciprocal, respectful, compassionate, and personal, such as those that may typically be found in companion animal relationships [157,168,169]. While many participants used companion animals as a common example, participants described a close affinity or shared experiences with animals in other categories, such as wild snakes, pigs, chickens, geese, sheep, wild birds, animals in biomedical research, and so on. This type of closeness is not necessarily limited to physical closeness but is characterised by an authentic sense of moral concern and compassion which recognises the animal as an individual with their own agency and interests. In intimate relationships, humans and animals, via a process of mutual interactions and intuitive approaches, develop a common language of understanding that may be embodied as well as vocal.

Importantly, our moral concern may extend beyond the individual to include other individuals, realising the potential of companion animals as ambassadors for other animals. Participants noted that these relationships therefore deserve wider cultural and academic recognition. Amiot, Bastian, and Martin (2016) have similarly stated that human/animal relationships have always been central to human existence [170]. These relationships have existed for a very long time; however, their importance and prominence have been underestimated and hidden as a result of the so-called “species-barrier” [168,171]. Levinson (1982) suggested that the potential for living at peace with animals lies in the investigation of these human–animal relationships, emphasising the importance of incorporating both intuitive and scientific methods: “The intuitive method looks at an animal as a teacher and friend, while the scientific method looks at an animal as an object of curiosity.” (p. 284).

This influence of closeness with companion animals as well as the positive relationship between increasing wealth, urbanisation, and concern for wild animals (feelings of mutualism) has been explored by several authors. Manfredo et al. (2020:1) questioned the suggestion that modernisation is the most prominent factor in favouring mutualist values (values that regard wild animals as individual fellow beings in a common social community) over dominance values (values that emphasise domination over wild animals). The authors argue that because modernisation has generally contributed to furthering the physical distance between humans and wild animals, there must be other moderating factors contributing to increased feelings of mutualism. Their results demonstrated that rising anthropomorphism is strongly associated with increasing mutualist values and could therefore partially explain why people in urbanised areas are increasingly regarding wild animals as deserving of rights similar to humans [172]. The authors discussed their results in the context of the Australian study results of Franklin (1999, 2007), who reported a significant positive and inclusive shift in human attitudes to animals as a function of the rising numbers of human relationships with companion animals [156,157].

In terms of concerns for the plight of farm animals, the potentially powerful influence of farm animal ambassadors has therefore become a topic of increased focus. This work has been conducted in sanctuaries that specifically aim to actively foster human connections with rescued farm animals, rather than passively displaying them as objects of human entertainment. A Faunalytics (2020) study reported the significant positive impact that physical/emotional contact and closeness with rescued farm animals had on visitors [173]. At these sanctuaries, visitors are given the unique opportunity to experience rescued farm animals as living, feeling individuals, each with their own story to tell. The stories combined with the closeness with individual rescued farm animals elicited feelings of heightened empathy and compassion for farm animals generally [174]. As a consequence, visitors felt compelled to make significant life changes as a gesture of solidarity or sympathy with farm animals. These findings are consistent with research that shows that people who include animals in their moral sphere are also more likely to extend prosocial behaviours towards them [158].

**Let animals speak.** Participants discussed the need to incorporate the subjective experiences of animals as individuals and persons for a more complete understanding of animals by amplifying animal voices. Amplification as much as possible attempts to avoid speaking for animals, focusing instead on enhancing animal voices or giving their voice a platform. They described this concept as advocacy and scientific approaches that recognise and promote the powerful agency of animals, giving animals a voice and a central place. Participants discussed alternative approaches to accessing and exploring *animal voice* and language, as demonstrated in other academic disciplines as well as various cultural and visual media. In *Slithering Stories we Live by: Animal Educators’ Construction and Enactment of Positive Snake Narratives*, Rosenfeld (2021) incorporated the concept of critical anthropomorphism to consider snake voices, demonstrating how animal voices can be empowered without their direct involvement. Via ethnographic methods, they explored how social setting, location, personal factors, and the stories told combine to shape conceptualisations of “snake” [175]. Rosenfeld (2021) demonstrated that negative perceptions of “snake” can be reshaped using these same mechanisms to improve human attitudes and reduce the influence of myths that increase our aversion towards and stigmatisation of certain animals.

This potential for empowering animal voices via public education and storytelling was illustrated by the participants who were involved in advocacy for typically stigmatised and *contextually voiceless* animals, such as snakes or animals categorised as “pests”. Via these alternative approaches, the animals themselves become the subjects with space to unfold their personalities, creating their own realities and allowing their *voice* a central place in our narratives. The unexpected and novel framing present a different perspective of animals, prompting us to consider the animal perspective.

Other studies have similarly attempted to address human–animal relationships with animals as the subjects, either in terms of species-specific or inter-species communication [65,176,177,178,179,180,181,182,183] or by presenting issues from the nonhuman point of view [184,185,186,187]. While participants noted that novel approaches should not exclude the attention to species-specific biology, life histories, behaviours, abilities, and communication, their insights suggested that it is via the additional incorporation of the subjective dimensions of animal wants and wills that animals can transcend from inanimate background props to become central, living individuals free to assert their voice and agency in a world shared with humans.

Importantly, participants remarked that we also need to consider to what extent we can assume a right to involve ourselves in animal lives, including issues related to animal consent. We need to carefully evaluate the ethical implications of involving animals, and if we do involve them, we must practice “epistemic humility” (acknowledging what we do not know about animals), echoing the concerns of Meijer and Bovenkerk (2021) (p. 50). At the same time, if we have a limited understanding of animal communication, it may limit our understanding of animal wants and interests. We have, for example, not been asking the right questions, as in cases where we ask questions about animal choices in settings where their choices are significantly limited [67]. Similar ideas for ethical approaches are suggested in studies that emphasise researching with animals as subjects as an attempt to disrupt the human tendency to speak for animals. For example, Barrett et al. (2021) suggested exploring intuitive interspecies communication (IIC) by using specialised animal communicators: people who have developed specialised skills through direct experiences with animals and as such, learnt to understand and interpret animal communication.

Our participants described similar respectful and compassionate approaches that they had used in their own work as well as future potentials for including these approaches in science, advocacy, and the political representation of animals. They also listed common themes related to reciprocal interactions and intimacy with animals, as well as respectful recognition of animal agency and personhood. Meijer (2021) discussed the potential for such ethical exploration of animal political agency and voice in sanctuaries like VINE, which offers refuge to escaped or rescued animals [188]. VINE challenges perceptions of animals as helpless and voiceless victims by encouraging and actively involving animal residents in community decisions. This requires that human staff put aside notions of themselves as hosts, rulers, and rescuers to engage in processes of interaction with the animal residents on their own terms. As a contrast to sanctuaries that advocate to encourage veganism, VINE acts as a model for improved interspecies relations, fostering connections with larger social justice movements and working towards systemic changes in our political and economic structures [188].

## 5. Conclusions

This study is the first to have explored themes related to animal representation in a cohort of participants from a variety of relevant expert and otherwise skilled backgrounds. The resulting theme, *animal voice*, and its subthemes indicated that animals and their interests are currently challenged by the entanglement with human interests and anthropocentric power structures. This has rendered countless animals artificially and contextually voiceless and thus vulnerable to harm, exploitation, and misrepresentation.

The themes from this study have identified barriers to empowering animal voices and authentically representing their interests. Our interview data reflects scholarly calls to reframe the plight of animals as a social issue as well as reconsidering institutional and individual perceptions of *animal voice*. Our results reflect the growing consensus in the literature that animals have intrinsic worth, independent of how humans value them.

This implies we need to seriously consider and recognise animals as individuals with their own intrinsic worth, voice, and agency in both public and professional discourses related to animals. It furthermore requires that we consider animals with respect and compassion at both individual and species levels, recognising animals as individuals with their own agency, personalities, interests, wants, and wills.

Echoing the recommendations of Meijer (2019), the themes developed from our interviews point to the need to develop ethical working relationships with animals, which requires that we move away from anthropocentric notions of animals as objects and instruments to consider animals as subjects and agents of their own lives. This by no means implies we ignore scientific developments related to the physical, cognitive, and emotional abilities of animals, but rather that we seriously consider the growing calls to develop and incorporate novel methods that focus on moving away from old anthropocentric conceptions and conjectures of animals as voiceless. However, how these approaches should be conducted and if/when we can assume to interfere in animal lives need to be considered within moral, ethical, and philosophical frameworks to foreground the animal perspective.

Recognising *animal voice* is an important step if we are to improve our understanding of animal interests at both individual and species levels, enabling us to devise and implement better animal-informed decisions to empower and amplify animal voices.

### Notes on Methodology and Author Personal Perspective and Potential Influence

The authors’ values are an important driver of this research. While the lead author acknowledges that personal views to some degree may be reflected in conversations with interviewees and the resulting data analysis, the Grounded Theory methodology incorporates steps to reflexively consider researcher views. The interview process is a dynamic interviewer–interviewee interaction to encourage free and open dialogue, allowing for richer data sets. It is not the role of the interviewer to control the responses on the sole basis of extracting untainted data, as this could equally taint the data and/or become a hindrance to the natural conversational flow. As much as possible, we have attempted to avoid imposing our own views during interviews and during data analysis.

## Figures and Tables

**Table 1:** Thematic analysis, including subthemes and summary of subthemes.

Themes	Subthemes	Summary
Animal Voice	Animals do/do not have a voice	Animals already speak/communicate Artificial voicelessness
		Sometimes animals need us to speak for them: contextual voicelessness
		Knowledge deficiency of animal language and communication
		We are only just beginning to understand
	Human language constructs realities and paradigms	Misguided conceptions and messages; inconsistency, speciesism, anthropocentrism, othering.
		Scientific reductionism, ontological, and epistemological approaches to understanding *animal voice*
	Let animals speak	Listening Communication is more than spoken language: embodied and visual. Knowledge, intimacy, compassion, respect, reciprocity Amplify animal voices

## Data Availability

The data are unavailable due to privacy and ethical approval restrictions and conditions.

## Supplementary Materials

The following supporting information can be downloaded at: https://www.mdpi.com/article/10.3390/ani13203271/s1, File S1: Semi-structured interview schedule.
