# Supplementary material for: Listen to Us: Perceptions of Animal Voice and Agency
Source: Animals (Basel). 2023 Oct 19;13(20):3271. doi: 10.3390/ani13203271 (PMC10603673; doi:10.3390/ani13203271)
Supplement: Supplementary file 1 [file animals-13-03271-s001.zip › animals-2580052-supplementary.pdf]

Semi-structured interview schedule.

Introduction: I would like you to think of someone who is going to speak on behalf of animals to represent their interests. Does that make sense, or would you like me to clarify?

1. How do people who speak for animals speak for animals?

2. Can you tell me how they reliably present the animal's interest?

    PROMPT: How might they?

3. What experiences or relationship with animals would someone like this have?

    PROMPT: Does this person have any particular personal characteristics that help them speak for animals?

    PROMPT: How would they consider animals?

    PROMPT: How do you think they listen to animals?

4. What does it look like for an animal's interests to be represented?

5. Are there people who can speak for many animals?

6. Who tries to speak for animals, but fails?

7. What experiences do you have with listening to animals?

    PROMPT: What did this experience tell you about the ways animals communicate with us?

8. When you hear the phrase: 'Who can speak for animals?' – what comes to your mind?

9. Before we finish, is there anything else you would like to add, or are there any other questions you can think of that you would have liked to be asked?

For the study, we need to know a little bit about you:

- What is your background or relationship with animals?
- Age range (18 to 24) (25 to 34) (35 to 44) (45 to 54) (55 and over)
- Do you live in a rural or urban setting?
- What gender do you identify by? (or prefer not to say)
